# Overall review of curative impact and barriers of CAR-T cells in osteosarcoma

**DOI:** 10.17179/excli2023-6760

**Published:** 2024-03-06

**Authors:** Guilin Li, Hong Wang, Vafa Meftahpour

**Affiliations:** 1 Xinyang Vocational and Technical College, Xinyang Henan 464000 China; 2 Medical Immunology, Cellular and Molecular Research Center, Urmia University of Medical Sciences, Urmia, Iran

**Keywords:** CAR-T cell, osteosarcoma, HER2+, GD2

## Abstract

Osteosarcoma (OS) is a rare form of cancer and primary bone malignancy in children and adolescents. Current therapies include surgery, chemotherapy, and amputation. Therefore, a new therapeutic strategy is needed to dramatically change cancer treatment. Recently, chimeric antigen receptor T cells (CAR-T cells) have been of considerable interest as it has provided auspicious results and patients suffering from low side effects after injection that resolve with current therapy. However, there are reports that cytokine release storm (CRS) can be observed in some patients. In addition, as researchers have faced problems that limit and suppress T cells, further studies are required to resolve these problems. In addition, to maximize the therapeutic benefit of CAR-T cell therapy, researchers have suggested that combination therapy could be better used to treat cancer by overcoming any problems and reducing side effects as much as possible. This review summarizes these problems, barriers, and the results of some studies on the evaluation of CAR-T cells in patients with osteosarcoma.

## Introduction

### State of art

Osteosarcoma, a form of bone cancer that impacts children and young adolescents, has a survival proportion of 65-70 % for non-metastatic types and less than 20 % for metastatic types. According to studies, around 65-70 % of cases are non-metastatic while 20 % are metastatic (Hogendoorn et al., 2010[42]; Meazza and Scanagatta, 2016[73]). Likewise, in other types of metastatic tumors, the molecular mechanisms behind osteosarcoma metastases remain unknown. Despite efforts to treat the disease through various methods such as amputation, surgery, and chemotherapy, there have been no significant improvements, leading researchers to focus on developing new therapies. One promising approach being studied for treating osteosarcoma is cellular immunotherapy, which has shown potential in oncology (PosthumaDeBoer et al., 2011[95]; Wang et al., 2019[118]). 

As already mentioned, the current treatments for osteosarcoma have limited success rates, prompting researchers to explore new therapies with the highest efficacy and lowest toxicity. While surgical interventions and chemotherapy have been used, promising options for cancer patients include adoptive cell therapy (cancer vaccine, DC vaccine, and CAR-T cells), Innate Immunity, and immune checkpoint (ICP) inhibitors yield more acceptable results. Several studies show disappointing outcomes in cancer vaccines for OS sufferers (Emens et al., 2009[26]; Lutz et al., 2011[66]; Simons et al., 2006[108]; Sondak et al., 2006[111]). In line with the DC vaccine, research shows a limited impact on recurrent osteosarcoma (Himoudi et al., 2012[41]). ICP leads to suppressing immune response by expression on cells. Meantime, tumor cells could express the ICP on their cells to escape from the immune response (Wang et al., 2019[119]). Albeit ICP blockade shows promising results, it is required to evaluate more combination therapies to detect and overcome the related problem (Meftahpour et al., 2022[74]).

### Literature review

The classification of CARs is determined by the signaling compartments they utilize. The initial CAR design, known as the first generation, solely featured a CD3ζ tail. In contrast, the second and third-generation CARs include the addition of one or two co-stimulatory domains, respectively. Moreover, two other generations of CAR-cells (TRUCKs and Universal CAR-T cells) are discussed below. In brief, autologous T cells are assembled from patients and injected back after altering their genes to allow the expression of CARs on the donor T cells. Then, CARs target the specific protein on the surface of tumors and eliminate it. CAR-T cell therapy has revealed significant results in hematological malignancies but faces complexities in solid tumors due to several issues, such as trafficking issues, finite endurance, infiltration, unfavorable tumor microenvironments (TME), and on-target off-tumor. To solve these problems, some solutions are mentioned namely, finding specific targets for tumors or antigens that are associated with tumors (TAA) or lowly expressed in normal organs to reduce the risk of toxicities and to promote CAR-T cells to selectively eradicate the OS cells (Caruso et al., 2015[12]; Liu et al., 2015[62]). In essence, identifying an ideal antigen target for CAR-T cell therapeutic application is still a monumental challenging step, and researchers studied and reported many papers in order to solve this problem. 

Patients who received CAR-T cells to cure or mitigate their diseases experienced disparate side effects, ranging from mild to severe, such as cytokine release syndrome (CRS), hypotension, flu-like symptoms, dyspnea, and neurologic difficulties (Ye et al., 2018[125]). The main challenge for curing with CAR-T cells is off-tumor and T cell exhaustion side effects that have been considered. In this context, some researchers have offered the potency of combination immunotherapy, such as combining ICP and CAR-T cells to treat solid tumors with low cytotoxicity.

### Contribution

The purpose of this paper is to collect the results and the failure of CAR-T cells in OS patients. Researchers have faced many problems and challenges to achieve their results and treat patients with no hazardous side effects. As mentioned earlier, solid tumors present some problems that should be considered when we discuss the competent treatment of CAR-T cells since it has not been achieved as in homological malignancies, because of the origin of heterogeneity in solid tumors.

In addition, in solid tumors, there are barriers that restrict the effectiveness of the curative impacts of CAR-T cells such as tumor microenvironment, vascular barriers, and T-cell exhaustion. However, the lack of overview of CAR-T cell therapy and obstacles in OS patients urges us to discuss the factors that contribute to CAR-T cell effectiveness as well as strategies enhancing the cytotoxicity potential of T cells in this review.

## Target Antigen in OS

To reach better clinical symptoms with as low as possible side effects after injection, it is imperative to identify the target antigen for early diagnosis, improve the power of molecular targeted therapy, and prognosis osteosarcoma. In addition, these antigens allow us to manage one of the most critical problems after treatment with various other therapeutic applications; on-target off-tumor. To date, several specific antigens have been identified whose expression in the exterior of tumor cells makes them a potentially promising target.

### Human Epidermal Growth Factor Receptor 2 (HER2)

The HER family, consisting of four members, (HER1-4), is a group of tyrosine-protein kinases that act as both homodimers and heterodimers. Having bonded to their ligands (epidermal growth factor (EGF) and transforming growth factor-α (TGF-α)), numerous physiological or pathological reactions are observed. Regarding HER2, however, it does not attach to any specific molecule. Instead, HER2 works by combining with other members, creating a heterodimer. Cell proliferation and tumorigenesis are induced because of different signaling approaches triggered by this collaboration (Yarden and Sliwkowski, 2001[124]). Studies have shown that HER2-targeted therapy has been effective in treating breast cancer, and it has also been found to be expressed in OS (Ahmed et al., 2009[3]; Scotlandi et al., 2005[105]). Therefore, investigating the therapeutic potential of anti-HER2 in curing or alleviating OS is worthwhile.

Studies by Ahmed et al. demonstrated that secreting immunostimulatory cytokines and proliferation as well as tumor regression and remission were observed after binding to OS cells expressing HER2 with HER2-CAR-T cells in cases of OS lung metastasis (Ahmed et al., 2009[3]). Similarly, other experiments showed significant antitumor responses of HER2-CAR-T cells in their studies (Park and Cheung, 2020[87]; Rainusso et al., 2012[98]). To evaluate the safety and efficacy of HER2 CAR-T cells in relapsed or refractory sarcoma, including OS, a clinical trial confirmed the stabilization of patients for several months with a dose of 1 × 108/m^2^ HER2 CAR-T cells, which was considered to be the optimal and well-tolerated dose. Three patients remained in remission after surgical removal of metastases. What is more, this therapeutic implication was the solution to infiltrating the dilemma of CAR-T cells as they demonstrated the ability to transport them to the tumor site and maintain HER-2-CAR-T cells at low levels for nearly two months. These findings suggest that HER2-CAR-T cells present be preferential treatment for OS by eliminating OS metastatic lesions that are considered the main reason for the death of OS sufferers (Ahmed et al., 2015[2]). However, there have been concerns about the safety of CAR-T cell therapy, as evidenced by a case report where some died from CRS following treatment with HER2-targeted CAR-T cells. One study designed a canine model to assess the cytotoxicity impacts of HER2-CAR-T cells and discovered lower cytokine production despite the similarity of the cytotoxicity impacts of canine HER2-CAR-T and human cells. The animal model could be worth studying the negative effects of CAR-T cell therapy. To address these adverse effects before clinical application, potential strategies including incorporating a suicide gene and inhibitory receptors into CAR-T cells have been evaluated (Mata et al., 2014[71]). HER2-CAR-T cell, by producing immune stimulatory T helpers (Th1) cytokines, leads to eradicated OS cells *in vitro* (Ahmed et al., 2009[3]).

### Disialoganglioside (GD2)

Disialoganglioside (GD2) is the cell membrane glycolipid that is being studied as a significant target in CAR-T cell application since the rate of expression of GD2 in OS is far higher than in healthy organs (Dobrenkov et al., 2016[25]; Lloyd and Old, 1989[63]; Navid et al., 2010[83]). The correlation between GD2 and molecules, proteins and other factors for migration is revealed. For example, it is reported that invasion of OS cells occurs with overexpression of GD2 by focal adhesion kinase (FAK) (Shibuya et al., 2012[107]). To vitiate the inhibitory function of MDSCs, All-trans retinoic acid (ATRA) is considered the best option to boost the response of this therapeutic strategy. Targeting GD2 for OS treatment has shown promising results. Studies have demonstrated that GD2-CAR-T cells can effectively kill GD2+ sarcoma cells and induce tumor cell death in OS. However, there are still challenges to be addressed. For instance, Long et al. showed that all of the OS samples they investigated expressed GD2. Although they illustrated the potential of the third-generation GD-2 CAR-T cell in recognizing and lyse sarcoma cell lines *in vitro*, they emphasized the lack of ability of GD2-CAR T cells to control tumor growth in a xenograft tumor model with periosteal injections of OS cell (Long et al., 2016[64]). 

GD2-targeted CAR-T cells have shown limited antitumor effects in xenograft models and can be inhibited by myeloid-derived suppressor cells (MDSCs) and the upregulation of PD-L1 expression in OS cells leading to CAR-T cell apoptosis (Chulanetra et al., 2020[17]). Strategies to improve the efficiency of GD2-targeted CAR-T cells include combining them with immune checkpoint blockade and constructing bispecific CAR-T cells. Also, to vitiate the inhibitory function of MDSCs, All-trans retinoic acid (ATRA) is considered the best option to enhance the antitumor response of GD2-CAR-T cells (Long et al., 2016[64]). Park and Cheung approved the increased efficacy of GD2 CAR-T cell in antitumor effect after combining with anti-PD-L1 (Park and Cheung, 2020[87]). In particular, OS cells are able to adopt various strategies to escape immune surveillance to an unprecedented extent, such as expressing PD-L1 to bind its ligand and inducing apoptosis of GD2-CAR-T cells expressed PD-1, although the annihilation effects of the fourth generation of GD2-CAR-T in OS have been uncovered (Chulanetra et al., 2020[17]). Therefore, it is worth exploring the combination of GD2-CAR-T cells with other members of immune checkpoint inhibitors to block this pathway and enhance the overall survival of patients with OS. Additionally, epigenetic regulation of tumor antigen expression in OS may be a viable approach to enhance the effectiveness of GD2-CAR-T cells.

### Type I Insulin-Like Growth Factor Receptor (IL-11RA)

IL-11RA, a receptor for the cytokine interleukin 11, plays a key role in autoimmune demyelination and cancer prognosis. In the case of osteosarcoma, high expression of IL-11RA provides an opportunity for researchers to investigate it as a potential therapeutic target (Liu et al., 2015[61]). A study found that IL-11RA overexpression in osteosarcoma patients was associated with increased tumor growth and aggressive metastasis (Huang et al., 2012[47]). Animal and human models have shown promising results with IL-11RA-targeted CAR-T cell therapy, leading to regression of lung metastases in IL-11RA-positive patients. The underlying reason for the alleviation property of IL-11RA CAR-T cells lies in the production of IL-11Ra and IL-11 by OS which urges tumor proliferation, invasion, and metastasis (Lewis et al., 2017[56]; Onnis et al., 2013[86]). In further experiments, the expression of IL-11RA was observed in osteosarcoma cell lines and lung metastases, and the ability to eliminate OS by IL-11RA-CAR-T cells was found (Huang et al., 2012[47]). Likewise, it is advisable to combine with other therapies to enhance antitumor activity since all OS cells do not express IL-11RA.

### Fibroblast activation protein

Fibroblast activation protein (FAP) is a serine protease and in adult human tissues is found normally low, however, it increases during tissue remodeling or cancer (Park et al., 1999[88]; Puré and Blomberg, 2018[97]). FAP is primarily associated with cancer-associated fibroblasts (CAFs), which have a key role in TME by secreting growth factors and forming a physical barrier. As a result, CAFs limit the CAR-T cell infiltration and further experiments to evaluate CAFs inhibitors in OS as well as other solid tumors are required. Inhibiting CAF has been recognized as a therapeutic strategy, and targeting FAP has shown promise in controlling tumor progression in preclinical models (Brennen et al., 2012[8]; Kakarla et al., 2013[49]). While there have been some studies on FAP expression in stromal fibroblasts in malignancies, its role in osteosarcoma (OS) has not been extensively investigated (Garin-Chesa et al., 1990[36]). However, Zhang Li et al. reported a higher rate of FAP expression in OS and its correlation with clinical pathological characteristics, proliferation, migration, and invasion of osteosarcoma cells (Zhang et al., 2020[128]). Studies of FAP expression in tumor cells demonstrate that FAP may serve as a potential diagnostic and a promising therapeutic target for OS (Crane et al., 2023[20]). 

### Type I Insulin-Like Growth Factor Receptor and Receptor Tyrosine Kinase-Like Orphan Receptor 1

From the outside looking in, tumors adopt distinct strategies, for example using receptors to migrate, invade, and metastasize, like type 1 insulin-like growth factor receptor (IGF1R) and the tyrosine kinase-like orphan receptor 1 (ROR1). Over-expression of ROR1 and IGF1R has been shown in solid tumors, such as OS and Ewing sarcoma (Cao et al., 2014[10]; Zhang et al., 2014[129]). The interactions between IGF1R and its ligand exhibit varying functions including inhibiting apoptosis, promoting synthesis of protein, and stimulating cancer invasion and metastasis (Hua et al., 2020[46]). In mice xenograft models, IGF-1R and ROR1 CAR-T cells targeted sarcoma, led to reduced tumor progression and improved clinical outcomes (Brennen et al., 2012[8]). Although IGF1R and ROR1 CAR-T cells have been tested in many cases, many of them are still in the early stages of clinical investigation. However, IGF1R and ROR1 may also be good targets for bone tumor durability and continuance in a sarcoma confined to a specific area (Huang et al., 2015[48], Wagner and Maki, 2013[116]). Studies have also indicated that ROR1 may be a promising therapeutic target to delay the metastasis of OS, as it was found to significantly inhibit the journey of OS cells. Additionally, anti-ROR1-CAR-T and anti-nti-IGF1R-CAR-T cells have demonstrated anti-tumor activity against sarcomas *in vitro *and have effectively suppressed sarcoma surge in both localized and scattered pre-established xenotransplantation models (Huang et al., 2015[48]).

However, the ideal TAAs are expressed as low as possible in healthy organs and, in contrast, highly expressed in tumor cells. Regarding this mindset, it is crucial to consider the ROR1 target therapy as ROR1 expresses in normal tissues, in particular in the gastric antrum. Nevertheless, researchers have developed a specific approach to protect the body tissues during target tumor cells, which shows promise in addressing off-target toxicity.

### B7-homolog3 (B7-H3)

Member of the B7 co-stimulatory molecule family, B7-H3 (also known as CD276) acts as an immune checkpoint protein that interacts with checkpoint markers like CTLA4 and PD-1. There is limited knowledge about the specific role of B7-H3, and numerous studies have provided varying information on its function. Obviously, B7-H3 is a protein that expresses low levels in healthy tissues, and in contrast, over-expression on solid tumors, such as OS and ES has been associated with tumor aggressiveness and metastasis (Wang et al., 2013[117]). B7-H3 inhibits the B7-CD28 superfamily that caused the inhibition of the proliferation and activity of T cells (Picarda et al., 2016[90]). Like all target antigens mentioned earlier, the expression of B7-H3 leads to promoting tumor cell invasion and decreases overall survival. Surprisingly, a previous study has observed the expression of B7-H3 on normal tissues in response to inflammation (Veenstra et al., 2015[114]). B7-H3 CAR-T cell therapy is a promising therapeutic application against primary and metastatic OS, as achieves notable clinical improvement. B7-H3 CAR-T cells can eradicate OS tumor cells and thus prolong survival and bring significant clinical improvements compared to a control group and on the other hand, almost all treated metastases patients with this therapeutic application, survived longer than untreated patients (Majzner et al., 2019[70]). Xenograft model studies have demonstrated the beneficial effects of B7-H3-CAR-T cells in terms of killing OS cells (Majzner et al., 2019[70]). Also, decreasing tumor size and improving health conditions with B7-H3-CAR-T cells were reported to approve the efficacy of B7-H3 target therapy (Murty et al., 2020[82]). In addition, B7-H3 is involved in several signaling pathways, including JAK/ STAT and PI3K/AKT/ mTOR, to promote tumor cell migration and invasion. Consequently, hindering such a pathway resulted in the cytotoxicity ability of anti-B7-H3 CAR-T cells (Li et al., 2017[59]; Nunes-Xavier et al., 2016[84]). Therefore, the combination therapy of B7-H3-CAR-T cells with other treatments could be considered as a cooperative curative way for OS. MGA 271 (Enoblituzumab) is a humanized IgG1 monoclonal antibody targeting B7-H3 to be scrutinized as an attractive option for immunotherapy. Powderly et al. reported evidence of anti-tumor activity of MGA 271 across several tumor types without any dangerous toxicity in early-phase clinical trials (Trial Registration: NCT01391143) (Powderly et al., 2015[96]). In the meantime, Loo et al. support the high efficacy of MGA 271 in both acceptable safety and antitumor activity (Loo et al., 2012[65]). To date, however, MGA 271 is not being widely studied in bone cancers which is required not to be neglected. The results of a phase 1 study involving pediatric patients and the drug MGA 271 were completed in May 2019, but the final findings have not been made available to the public (Desantes et al., 2017[24]; Smrke et al., 2021[110]).

### CD44v6

Another therapeutic target is a cell surface glycoprotein with roles in cell proliferation, differentiation, migration, and angiogenesis named CD44. The isoform 6 of adhesive receptor CD44 (CD44v6) is also known as a biomarker of tumorogenesis, whose expression in OS has been observed and correlated with poor prognosis and metastasis rates (Wang et al., 2018[120]; Zhang et al., 2015[130]). What is more, CD44v6 regulates the extracellular matrix, promotes cell motility, and suppresses tumor apoptosis. As a result, its expression correlates with the high-risk factor and attracts researchers to examine CAR-T cell therapy. Porcellini and colleagues demonstrated the efficacy of CD44v6 CAR-T cells by controlling the tumor growth in the patients with lung and ovary adenocarcinoma in mice models (Porcellini et al., 2020[92]). Meanwhile, Wrana et al. indicated the link between high expression of CD44v6 and high dual expression of CD44v6 with the cell adhesion molecules integrin α2β1 and PD-L1 in early recurrence after hepatectomy in liver metastatic patients (Wrana et al., 2022[122]). As a matter of fact, the accurate evaluation of the curative property of CD44v6 CAR-T cells in OS patients is far because of the lack of evidence. Hence, more experiments should be considered.

### Natural Killer Group 2D (NKG2D)

NKG2D is a receptor found in natural killer (NK) cells and T cells that play a crucial role in immune responses during several diseases, ranging from cancer to infections. Expression of NKG2D in either osteosarcoma or infected cells and malignant tissue has been reported (Fernández et al., 2015[32]; Lehner et al., 2012[55]). The interaction of NKG2D and its ligand (NKG2DLs) leads to cell activation, the release of cytotoxic granules, and then cell apoptosis. Hence, it is not surprising that the involvement of NKG2D in cancer sufferers has been increasing interest study. Regarding OS, the significant ligand for NKG2D is commonly expressed, as MHC class I chain-related molecule A (MICA). Compared to benign tumors and normal bone tissue, OS exhibits higher levels of MICA expression. Restoring the expression of NKG2D receptors on immune effector cells could potentially be used as a therapeutic approach for treating human OS. Recently, the second-generation NKG2D-directed CAR-T cells against osteosarcoma have been explored. Results have shown increased response activity against OS cells and enhanced overall survival without damaging normal cells (Fernández et al., 2017[31]). Precisely, Fernández et al. expanded the use of NKG2D to T cells by creating NKG2D CAR-redirected memory T cells, which enhanced their ability to kill OS cells in laboratory settings compared to T cells that were not transduced. Additionally, *in vivo*, the growth of tumors was inhibited, and survival rates were improved in mice that received NKG2D CAR-redirected memory T cells (Fernández et al., 2017[31]). Importantly, these modified T cells did not exhibit any harmful effects on healthy cells or chromosomal abnormalities caused by the lentiviral transduction process. Since the killing properties of NKG2D CAR-T cells on cancers, such as leukemia (Li et al., 2022[57]), colorectal (Deng et al., 2019[23]), and cervical cancer (Zhang et al., 2020[131]) are revealed, it is expected an increased response in OS patients.

### Activated leukocyte cell adhesion molecule (ALCAM, CD166) 

ALCAM belongs to the immunoglobulin superfamily and after binding with its ligand, CD6, various biological activities, ranging from neuronal outgrowth, and hematopoiesis to inflammatory responses are observed (Swart, 2002[113]). Regarding tumorigenesis of ALCAM, some experiments approved the role of ALCAM in breast cancer, prostate cancer, glioblastoma, colorectal, and OS (Federman et al., 2012[28]; He et al., 2023[40]; Kijima et al., 2012[50]; King et al., 2004[52]; Kristiansen et al., 2003[53]). For the first time, Wang et al. demonstrated that CD166 CAR-T cell is a novel approach for the treatment of OS (Wang et al., 2019[118]). Notably, ALCAM is not only expressed in tumors, but also is expressed on normal cells, including epithelial cells, fibroblasts, and neurons. Therefore, this experiment was overshadowed by the safety issues related to off-tumor toxicity and it is required for further studies to solve.

### Chondroitin Sulfate Proteoglycan 4 (CSPG4)

Chondroitin sulfate proteoglycan (CSPG)4 is a transmembrane proteoglycan with low expression in healthy tissues and high expression in tumor cells (Benassi et al., 2009[5]), which have a central tumorigenic role responsibility. As high CSPG4 expression correlates with short survival and metastasis-free survival, the CSPG4 targeting strategy is considered an attractive therapeutic application. In an experiment conducted by Riccardo et al., CSPG4 is an ideal anticancer target therapy in OS. However, further studies are needed to evaluate the advantages and disadvantages of this therapeutic strategy (Riccardo et al., 2019[101]).

### Erythropoietin-Producing Hepatocellular Receptor Tyrosine Kinase Class A2 (EphA2)

It is an axiomatic fact that target receptors that are expressed in OS and lowly expressed in normal bone tissues attract researchers' attention, including EphA2, a tyrosine kinase receptor (PosthumaDeBoer et al., 2013[94]). The correlation between overexpression of EphA2 and carcinogenic signals, angiogenesis, and tumor invasiveness in OS is approved (Fritsche‐Guenther et al., 2010[34]; Garcia‐Monclús et al., 2018[35]; Sainz-Jaspeado et al., 2013[103]). In the meantime, anti-EphA2-CAR-T cells *in vitro* and *in vivo* experiments were evaluated in OS by Hsu et al. with a promising result. Interestingly, anti-EphA2-CAR-T was tested in the aggressive metastatic OS model and showed the ability to kill and clear metastatic tumors in the lung and liver of mice (Hsu et al., 2021[45]). Evidently, the chances of tumor immune escaping are high due to an increase in EphA2-negative cells for no reason. To solve this problem, it is required to evaluate the combination of EphA2-CAR-T cells with ICP blockade or chemotherapy (Table 1(Tab. 1); References in Table 1: Anderson et al., 2016[4]; Chulanetra et al., 2020[17]; Murty et al., 2020[82]; Olmos et al., 2010[85]; Park and Cheung, 2020[87]; Pollak, 2012[91]; Schwartz et al., 2013[104]).

## Boosting Approaches

The effectiveness of CAR-T cells in various diseases is reduced by obstacles, namely inadequate movement of CAR-T cells and the depletion of TIL. One commonly used approach involves the use of T-cell inhibitory receptors. Numerous studies have been conducted to address these challenges, but combining different therapies is considered a preferable strategy by researchers. This article explores several immunosuppressive pathways that can restrict the maximum benefits of adoptive CAR-T cell therapy and strategies to overcome these problems in patients with sarcoma.

### Tumor Micro Environment's impacts

The tumor microenvironment contains various elements that hinder the effectiveness of CAR-T cells in fighting tumors. The osteosarcoma microenvironment is a unique and intricate osseous environment that includes osteoblasts, stromal cells, vascular cells, immune cells, and mineralized extracellular matrix (Corre et al., 2020[19]). It is worth mentioning that osteosarcoma-formed osteoid bone tumor matrix inhibits the access of CAR-T cells to OS tumor sites. The production of an OS-like matrix is influenced by different cytokines and growth factors, including transforming growth factor-beta1 (TGF-b1), fibroblast growth factors (FGFs), and members of the wingless MMTV integration site family (WNTs). In normal circumstances, the functioning of bones relies on the harmonious interactions among these components, cell communication, and signaling for maintenance (Corre et al., 2020[19]). However, OS cunningly exploits these microenvironments to sustain its survival and proliferation, posing significant challenges to the efficacy of CAR-T cell therapy. However, this suppression is largely dependent on the specific conditions within the tumor microenvironment. When CAR-T cells are removed from the tumor, their anti-tumor functions are restored (Moon et al., 2014[80]). This indicates that inhibiting immunosuppression or modifying the immunosuppressive tumor microenvironment could potentially enhance the function of CAR-T cells and provide new opportunities for therapy. Immunosuppressive cytokines, such as TGF-β or IL-10 secreted by both pro-tumoral immune cells can conquer the immune response.

The functions of TGF-b1 range from promoting bone formation to inhibiting the differentiation of bone marrow mesenchymal stem cells into osteoblasts and the mineralization of mature osteoblasts during the late stage of osteoblastic formation (Maeda et al., 2004[69]). In the context of OS, TGF-b plays a role in tumor growth and metastases (Verrecchia and Rédini, 2018[115]). As demonstrated by Lamora et al., TGF-b is more expressed in the serum of OS sufferers than in healthy individuals (Lamora et al., 2014[54]). Therefore, TGF-b receptor target therapy may provide a promising opportunity for the treatment of OS. To repolarize the tumor microenvironment, various strategies have been tested, including armored' CAR-T cells or 'TRUCKs' (T cells redirected for universal cytokine killing). To enhance the cytotoxicity effects of CAR-T cells in an immunosuppressive microenvironment, armored-CARs and TRUCKS release pro-inflammatory cytokines, like IL-12, which are expressed in CAR-T cells leading to tumor regression (Chinnasamy et al., 2012[14]). This effect is mediated by changes in the number and function of myeloid cells present in the tumor. The constitutive signaling of IL-12 enhances T-cell cytotoxicity and cytokine secretion, promoting resistance against immunosuppression by regulatory T cells (Tregs).

FGF is platelet lysate's growth factor found in bone matrix which has a crucial role in skeletal development, regulation of chondrogenesis, osteogenesis, and bone fracture healing (Meftahpour et al., 2022[75]). The correlation of FGF and its ligand (FGFR) urges tumor progression (Zhou et al., 2016[133]) and a high level of FGFR1 was detected in OS patients (Weekes et al., 2016[121]). If the signal transmission of FGFR1 is blocked, this can prevent metastasis. The problem, however, is the expression of FGFR1 in CD4+ T cell subsets. By producing IL-2 through FGFR1+ to activate and proliferate T cells, the interaction of FGF and FGFR creates a main obstacle to CAR-T cell therapy (Byrd et al., 1999[9]; Coppola et al., 2020[18]).

WNT, a group of secreted glycoproteins, plays a role in bone development and dynamic homeostasis (Matsuoka et al., 2020[72]). The WNT signal stimulates the proliferation and metastasis of OS. In a study conducted by Goldstein et al. using a preclinical model, inhibition of the metastasis of osteosarcoma by using a monoclonal antibody, Dickkopf-1, that blocks the WNT signal inhibitor (Goldstein et al., 2016[37]). This approach is expected to hinder tumor growth by reducing the activation of WNT transcription. However, it is important to note that the WNT pathway is complex due to its effects on the differentiation of stem cells, and tissue regeneration as well as regulates the normal functioning of healthy cells (Singla et al., 2020[109]). What is more, WNT has a downstream impact on the signaling approach of MET, a tyrosine kinase receptor that is overexpressed in OS to pave the of conversion of osteoblasts into osteosarcoma cells (Kim et al., 2013[51]; Patane et al., 2006[89]). MET is assumed a potential target for CAR-T cells as resulted in strong cytotoxicity against solid tumors (Yuan et al., 2021[126]).

### Tandem CARs (TanCARs)

Tumor recurrence due to the loss or alteration of the marked antigens is often detected in tumor cells (Sotillo et al., 2015[112]). As a result, tumor recurrence is mainly prevented if two or more TAAs are fully targeted since the chances of failing both antigens are much lower than a single antigen. To clarify, an experiment was conducted to evaluate the bispecific CAR, TanCAR, which united two disparate scFvs in tandem to a receptor of transgenic so that TanCAR is able to freely move. This study revealed the synergistic effect of CAR which leads to improved cytotoxicity ability of CAR-T cells (Bielamowicz et al., 2018[6]; Grada et al., 2013[38]).

The significant advantage of TanCAR compared with one marked antigen CAR or a combination of 2 single marked antigens CARs, is its ability to prevent the escape of tumor cells and improve anti-tumor effects with less toxicity. Albeit TanCAR has attracted much attention to be studied for cancer treatments, there is no report for OS yet (Miao et al., 2021[77]).

### Universal CARs

**Figure 1:**
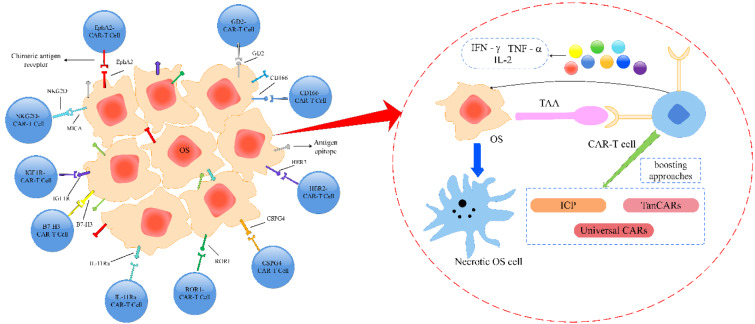
Combining different therapies is important for improving the overall survival of patients with osteosarcoma. One promising approach is the use of CAR-T cells, which are engineered to target specific tumor-associated antigens (TAAs) and release factors that kill OS cells. However, there are several challenges that need to be addressed, such as difficulties in infiltrating the tumor and tumor microenvironment. Additionally, there is a risk of harmful effects on healthy cells due to off-target toxicity. Therefore, combination therapy approaches are necessary to overcome these barriers and enhance the effectiveness of CAR-T cell therapy in OS patients.

To increase the power of CAR to recognize different antigens without requiring modification of the T-cell, the CARs of the fifth-generation, known as universal CARs, utilizing “third party” intermediate system to isolate the antigen-binding domain of the CAR from the T-cell signaling unit is designed (Ma et al., 2016[67]; Zhao et al., 2018[132]). In essence, the goal of universal CARs is to minimize tumor cell escape. There are two types of universal CARs: BBIR CAR and SUPRA (split universal and programmable) CAR. The system of BBIR CAR consists of two components, dimeric avidin (BBIR) which connects to the membrane of T cells, and biotin which links to a particular antigen of molecules. These components are combined non-covalently to build a targeted relation between T cells and antigens, ultimately promoting these cells' response. The anti-SUPRA CAR organization (Cho et al., 2018[15]) also consists of two components, a T cell universal receptor with a leucine zipper adaptor (zipCAR), and a single-chain variable fragment with a leucine zipper adaptor (zipFV) that targets TTA. The combination of zipCAR and zipFV enhances the sensitivity of T cells, leading to T cell activation and target TAAs. The Universal CARs are considered to be the ideal CARs and it is envisaged for further clinical trials for cancer diseases, like OS (Figure 1(Fig. 1)).

### iCARs

When it comes to preventing off-target side effects after CAR-T cell therapy, inhibitory CARs (iCARs) are one of the strategies to approach to enhance the general curative effect of CAR-T cells. iCARs, could support normal cells, by targeting TAA, not the antigens that are expressed in healthy tissues. After binding with normal cells; iCARs produce inhibitory signals to suppress T-cell function, hence the normal cells are protected. Therefore, iCARs are able to distinguish tumor cells from normal cells (Fedorov et al., 2013[29]). Evidence has shown the feasibility of iCARs harnessing natural T cell inhibition exerted by immune checkpoint (PD-1 and CTLA-4) to survive normal cells (Fedorov et al., 2013[29]).

### Immune checkpoint inhibitors

By combining immune checkpoint blockade (ICB) with CAR-T cells, we can see promising results with a low degree of adverse effect (Chong et al., 2017[16]). The combination of ICB with CAR-T leads to express the CCR2 receptor (to enhance homing to the tumor), IL12 (to enhance cytolytic activity and T cell survival), and IL7 (to enhance memory differentiation) (Ren et al., 2017[100]). Indeed, the FDA approved the targeting of these inhibitory molecules by ICB are ipilimumab and nivolumab, which led to the enhancement of anti-tumor response and clinical responses in patients (Ahmed et al., 2015[2]). 

**Figure 2:**
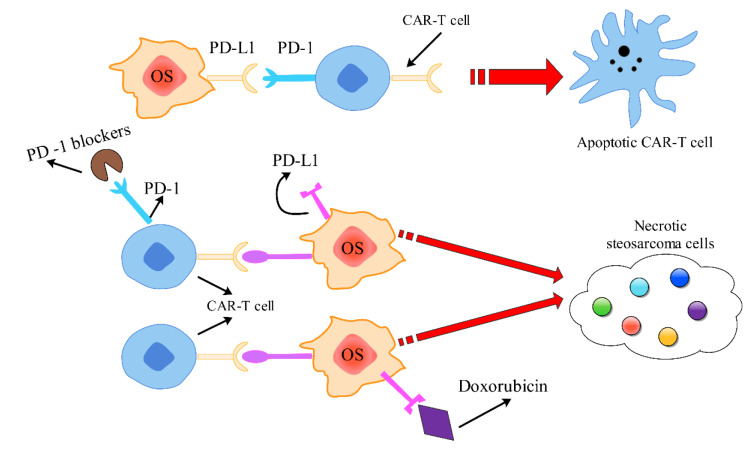
The presence of immune checkpoints, such as PD-L1 and CTA-4, on the surface of osteosarcoma cells allows them to evade detection by CAR-T cells, leading to their apoptosis. To address this issue and enhance the binding of CAR-T cells to tumors, researchers have explored the use of immune checkpoint inhibitors that target these immune checkpoints on CAR-T cells. Another approach involves combining chemotherapy drugs, like Doxorubicin with ICPs that are expressed in osteosarcoma cells, which has shown promising outcomes.

Osteosarcoma cells express immune checkpoints, such as PD-L1, CTA-4, and to name a few others, on their surface to escape immune surveillance by inducing apoptotic of CAR-T cells. In order to prevent apoptotic and bind CAR-T cells to tumors, connecting ICP inhibitors, such as pembrolizumab or nivolumab and so forth, with ICP expressing on CAR-T cells or chemotherapy drugs, such as Doxorubicin, with ICP expressing on OS cells have been examined and given promising results (Figure 2(Fig. 2)).

### Co-stimulatory molecules 

CAR-T cells have exhaustion markers that can limit their functions, such as co-stimulatory molecules like CD28 and 4-1BB, that drive CAR-T exhaustion and limited persistence, hence they represent potential therapeutic targets (Zhang et al., 2017[127]). In summary, the survival of the property of anti-tumor CAR-T cells relies on co-stimulatory signals. Considering these signals when designing synthetic T cells and finding the optimal co-stimulatory signal(s) for CAR design is highlighted. Meanwhile, studies have shown that disrupting co-stimulatory signaling by inducing mutations in specific amino acid remains within the CAR's CD28 signaling domains can boost the persistence and durability of CD28ζ CAR-T cells (Honikel and Olejniczak, 2022[44]). An experiment supported the reduction of CAR-T cell exhaustion by alteration of the proximal tyrosine (YMNM → FMNM) and the middle proline (PRRP → ARRA) signaling domains that result in decreasing the expression of exhaustion-related genes, NFAT (Boucher et al., 2021[7]). However, further research is critical to fully grasp the interaction between CAR and co-stimulatory signals to cure the serious dilemmas of exhaustion in T-cell therapy.

### Other factors

In line with the continuous progress of technology and medical science, therapeutic applications to cure cancer patients have significantly evolved. To date, many combination approaches to enhance the method of CAR-T cell curative effects have been tested. The combination of CAR-T cells with tumor vaccination therapy (Reinhard et al., 2020[99]); radiotherapy or chemotherapy (Feng et al., 2018[30]; Minn et al., 2019[79]); oncolytic viruses (Porter et al., 2020[93]); photothermal therapy (Chen et al., 2019[13]); CRISPR/Cas9 (Eyquem et al., 2017[27]), and nanotechnology (Ma et al., 2020[68]) are being evaluated worldwide by researchers.

## Conclusion

Currently, overcoming treatment resistance in OS remains a significant obstacle in the field of immunotherapy. Traditional therapies have shown limited success in treating localized tumors, but have not been effective in treating patients with advanced disease. Various approaches have been explored to enhance the survival of patients with advanced oncology, but none have yielded promising outcomes thus far. CAR-T cell immunotherapy reached a significant milestone in the treatment of cancer patients in recent years. This therapeutic application was evaluated in other cancers, which showed the power of CAR-T cells in treating the diseases, such as glioblastoma, Ewing sarcoma, and neuroblastoma. As discussed above, the general curative impacts of CAR-T cells are not restricted to TAAs and many factors are involved in decreasing or increasing the therapeutic application of CAR-T cells. However, there are other limitations that should be considered, such as cell cancer metabolisms. Precisely, the cytotoxicity effect of CAR-T cells is overshadowed by metabolic and nutrient-sensing mechanisms, such as glucose, prostaglandin E2, fatty acids, cholesterol, and so forth (Li et al., 2023[58]). Also, solid tumors produce lactate by the glycolytic pathway that suppresses the efficacy of human cytotoxic T lymphocytes by decreasing the cytokine release level or other mechanism (DeBerardinis and Chandel, 2016[22]; Fischer et al., 2007[33]). In addition, insufficient trafficking and infiltration of CAR-T cells are other topics to discuss. In other words, high endothelial venules (HEVs) are involved in T-cell infiltration and have a key role in tumor regression (Ager, 2017[1]). To enhance the infiltration of CAR-T cells to tumor site experiments, for instance, anti-angiogenic therapy targeting VEGF, CD276, and combination with CAR-T cells are being evaluated (Daenen et al., 2009[21]; Yang et al., 2018[123]) and FAP-CAR-T cells reducing tumor fibroblast numbers and thus inhibiting tumor growth (Lim et al., 2020[60]; Moon et al., 2018[81]). Angiogenesis provides fertile ground for tumor growth and invasion by preparing oxygen and nutrients under normal physiological conditions. OS produces TGF-alpha and other pro-angiogenic factors to induce angiogenesis of endothelial cells through Prolyl hydroxylase-4 (PHD4) which is expressed in OS (Moon et al., 2018[81]). The new blood vessels are smaller and more sensitive than normal vessels, which has limited effects on T cell differentiation and CAR-T cells. One of the most challenging physical barriers in this therapeutic strategy is the Fibrotic extracellular matrix (ECM) which can be deteriorated by heparanase (HPSE). Meanwhile, CAR-T cells are engineered to express HPSE and have shown T-cell infiltration and antitumor activity (Min et al., 2017[78]). Mesenchymal stem cells (MSCs) have a variety of physiological functions from tissue repair and wound healing to suppressing immune response and supporting tumor progression (Caruana et al., 2015[11]). Some studies have indicated that modifying MSCs through genetic engineering can transform their pro-tumorogenic chronic profile into a supportive one, thereby improving the effectiveness of CAR-T cell therapy in targeting tumor sites (Meftahpour et al., 2021[76]; Ryan et al., 2005[102]). In one study, MSCs were manipulated to deliver IL-7 and IL-12 to the tumor sites that enhance CAR-T response and anti-tumor activity by changing the profile of chronic inflammatory Th2 into Th17/Th1 profile which leads to longer-lasting anti-tumor CAR-T cells response (Haabeth et al., 2011[39]; Shalapour and Karin, 2015[106]). In line with the side effects of CAR-T cell therapy, patients suffer from fevers, hypotension, hypoxia, and neurologic dysfunction due to CRS which is renowned for the on-target toxicity mode of CAR-T cells (Feng et al., 2018[30]; Hombach et al., 2020[43]). We can conclude that the efficacy of CAR-T cell therapy would be better by combination with another therapeutic strategy to overcome some barriers and factors that limit the treatment application and eradicate the tumor cells with as low as possible side effects. To achieve this point, we need further studies and more examination.

## Declaration

### Acknowledgments

We apologize that because of space constraints, we were unable to cite many studies. The authors declare that they are not supported with external funding.

### Funding

This research did not receive any external funding. All expenses related to this study were covered by the authors themselves.

### Availability of data and materials

The datasets used in this study are available upon reasonable request.

### Ethical approval

Not applicable.

### Competing interests

The authors declare no competing interests, whether of a financial or personal nature, that could influence the research or its interpretation.

### Patient consent for publication

Not applicable.

### Authors' contribution

Supervision, project administration, conceptualization: Guilin Li

Methodology, software, language review: Hong Wang

Validation, writing a draft preparation: Vafa Meftahpour.

## Figures and Tables

**Table 1:**
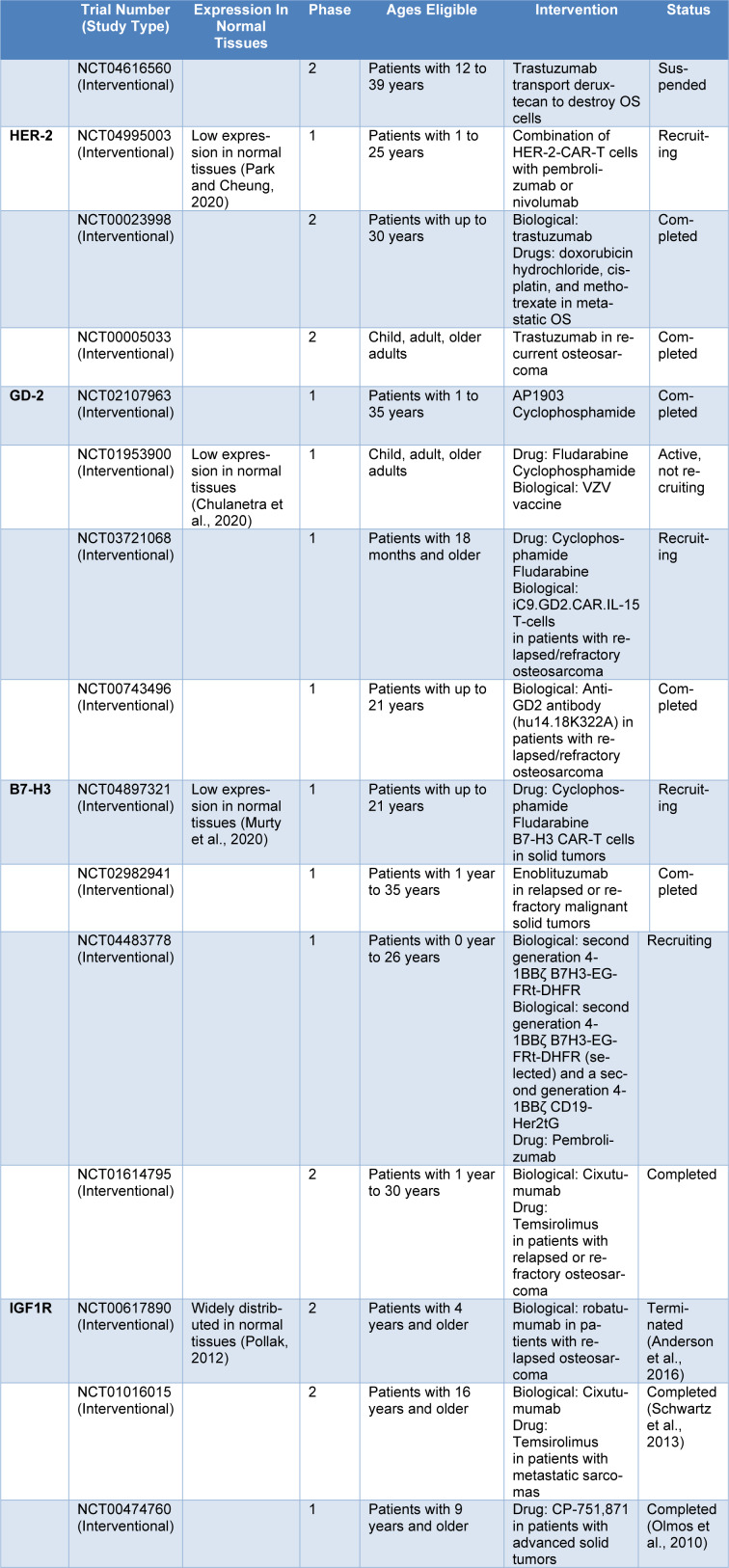
TAAs in osteosarcoma
